# Anomalous Halo Formation in an Arc-Melted ScNi-Sc_2_Ni Off-Eutectic Binary Alloy

**DOI:** 10.3390/ma9070584

**Published:** 2016-07-18

**Authors:** Kai Wang, Ming Wei, Lijun Zhang

**Affiliations:** State Key Laboratory of Powder Metallugy, Central South University, Changsha 410083, China

**Keywords:** anomalous halo, eutectic solidification, phase-field model

## Abstract

Diverse non-equilibrium eutectic structures have attracted numerous experimental and theoretical studies. One special type is the formation of a halo of one phase around a primary dendrite of another phase. In our experiments, it was occasionally observed that ScNi halos grow as dendritic morphology around the primary Sc${}_{2}$Ni dendrites in an arc-melted ScNi-Sc${}_{2}$Ni off-eutectic binary alloy. The formation of this anomalous halo structure was then well reproduced by employing quantitative phase-field simulations. Based on the phase-field simulation, It was found that (i) the large undercooling and growth velocity of the ScNi phase during solidification causes the formation of halos; and (ii) the released latent heat induces the recalescence phenomenon, and changes the solidification sequence largely, resulting in the anomalous halo structure in the Sc-34 at % Ni alloy.

## 1. Introduction

Alloys with eutectic microstructures are widely used as structural and/or functional materials in automobile, aerospace, electronic and other industries [1,2,3,4,5] due to their excellent comprehensive properties and performances. For off-eutectic alloys, their typical microstructure generally comprises not only coupled eutectics, but also dendrites of primary phase under equilibrium solidification. However, in real solidifications, which usually deviate from equilibrium state, diverse non-equilibrium eutectic structures appear and have attracted numerous experimental and theoretical studies [6,7,8,9,10]. One special type is the formation of a halo of one phase around a primary dendrite of another phase, as observed in several off-eutectic alloys, like Al-Si [11], Al-Cu${}_{2}$Al [12], Ni-Ni${}_{3}$Nb [8], Cr-Nb [7] and Fe-Fe${}_{2}$Nb [13].

**Figure 1:**
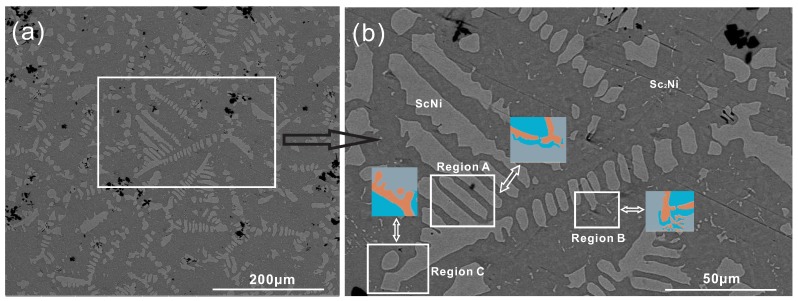
(**a**) Experimental microstructure of as-cast Sc-34 at % Ni binary alloy; (**b**) Enlarged view of the rectangular section in (**a**).

Occasionally, we observed an anomalous halo formation in an as-cast, off-eutectic Sc-Sc${}_{2}$Ni alloy, i.e., Sc-34 at % Ni in the present work. Pure nickel (99.99%) and pure scandium (99.99%) were used as the starting materials, and the Sc-34 at % Ni alloy was melted in an arc melting furnace (Physcience Opto-electronics Co., Ltd., Beijing, China) and placed in the copper mold, which was then cooled by the circulating water. The casting was carefully polished and the microstructure of Sc-34 at % Ni was characterized by means of scanning electron microscope (SEM). The result is shown in Figure 1. Figure 1b is an enlarged view of the rectangular section in Figure 1a. As can be seen in Figure 1b, the dark region presents the Sc${}_{2}$Ni phase, which is the first primary phase and grows as a coarse dendrite during solidification. The bright region presents ScNi grains, which nucleate and grow along the interface of the Sc${}_{2}$Ni dendrite and the remaining liquid phase. The ScNi phase grows as the second primary phase instead of the fine lamellar microstructure, and the (ScNi) dendrite even appears at the tip of the Sc${}_{2}$Ni dendrite. Moreover, at the arm of the Sc${}_{2}$Ni dendrite, the halo morphology is substituted by coarse-coupled eutectic and the lamellar eutectic structure nucleates and grows at the interface of the dendritic-like ScNi phase and the remaining liquid. As a result, all these characteristic features from the anomalous halo microstructure occurr in this as-cast, off-eutectic Ni-Sc alloy during solidification.

Several mechanisms have been proposed to explain the halo formation in off-eutecitc alloys during solidification. Sundquist et al. [14] contended that certain undercooling is necessary for halo formation. Kofler et al. [15] emphasized that halo formation relies on the relative growth rates at a given undercooling. According to Gigliotti et al. [9], halo formation is influenced by not only the growth velocities of the involved phases but also the undercooling and solute content. Nave et al. [6] stated that halo formation should be compatible with the diffusion fields of the primary phase during directional solidification. Actually, these mechanisms were proposed on the basis of their own experiments, and can be employed to explain the halo formation which entirely surrounds the primary phase during unidirectional solidification. During unidirectional solidification, the effect of latent heat can be ignored due to the large temperature gradient and thermal diffusion. However, the latent heat should be taken into account in the present Sc-Ni sample. That is because the latent heat in such a small sample can result in significant recalescence phenomenon, because the temperature of the remaining melt increases in a short period and the existent microstructure may be remelted. Therefore, the above-mentioned mechanisms cannot be utilized to explain the anomalous halo formation in the present Sc-Ni off-eutectic alloy.

Under this circumstance, the quantitative phase-field simulation was employed to reveal the mechanisms for the anomalous halo formation in the present ScNi-Sc${}_{2}$Ni off-eutectic alloy. It is well known that the quantitative phase-field simulation can not only reproduce the microstructure evolution during various materials processes but also reveal inherent physical natures [16,17]. Very recently, Wei et al. [18] performed a quantitative phase-field simulation of the microstructure evolution in A2214 commercial alloy during solidification by using the multi-phase-field (MPF) model, from which all the experimental features were nicely reproduced, and the physical mechanisms for some microstructure formation were also revealed. Ta et al. [19] predicted the microstructure evolution in different Ni-Al-Cr bond coat/substrate systems based on the quantitative phase-field simulation using the MPF model, and also investigated the effect of the temperature gradient. Based on the quantitative phase-field simulation, Wang et al. [20] reproduced the three-dimensional microstructure evolution of primary silicon in one hypereutectic Al-Si alloy by using the MPF model, from which diverse two-dimensional morphologies of primary silicon in experiments can be well explained. Consequently, the MPF model, which was already incorporated in the MICRESS (Microstructure Evolution Simulation Software) [21], was utilized here to reproduced the microstructure evolution in the present Sc-34 at % Ni alloy during solidification, from which the mechanisms for the anomalous halo formation can be then revealed.

## 2. Phase-Field Simulation

### 2.1. Phase-Field Model

Considering that the MPF model with phase and concentration fields has been frequently described in our previous work [18,19,20,22,23], it is not presented in detail here in order to save space. However, the temperature field is necessary to be introduced here in order to reproduce the microstructure in the present target alloy, and thus is presented in the following section. That is because the sample is relatively small and the temperature gradient can be negligible. Thus, the cooperative effect of the external heat extraction and the latent heat should be taken into account. According to the heat balance [24]:

(1) $$\begin{array}{ccc} \dot{q}dt & = & \frac{1}{V}\int_{V}\sum_{\alpha }d(H_{\alpha }\phi_{\alpha })dV \end{array}$$

The global heat extraction per volume $\dot{q}$ is equal to the total enthalpy change in the system. $H_{\alpha }$ is the specific enthalpy of phase *α*. The enthalpy change $d(H_{\alpha }\phi_{\alpha })$ can be separated into three parts: a temperature dependent term, corresponding to the heat capacity $c_{p,\alpha }$; a concentration dependent term caused by segregation or diffusion; and finally, the release of latent heat due to phase transformation.

(2) $$\begin{array}{ccc} d(H_{\alpha }\phi_{\alpha }) & = & \phi_{\alpha }\left(c_{p,\alpha }dT+\sum_{k}\frac{\partial H_{\alpha }}{\partial c_{k}}dc_{k}\right)+H_{\alpha }d\phi_{\alpha } \end{array}$$

When substituting Equation (2) into Equation (1), the temperature change dT can be evaluated as:

(3) $$\begin{array}{ccc} dT & = & \frac{1}{{\bar{c}}_{p}}\left(\dot{q}dt-\frac{1}{V}\int_{V}\sum_{\alpha }\left(\phi_{\alpha }\sum_{k}\frac{\partial H_{\alpha }}{\partial c_{k}}dc_{k}+H_{\alpha }d\phi_{\alpha }\right)dV\right)\\ & = & \frac{1}{{\bar{c}}_{p}}\left(\dot{q}dt-dL\right) \end{array}$$

with ${\bar{c}}_{p}=\frac{1}{V}\int_{V}\sum_{\alpha }c_{p,\alpha }\phi_{\alpha }dV$.

### 2.2. Phase-Field Simulation Parameters

**Figure 2:**
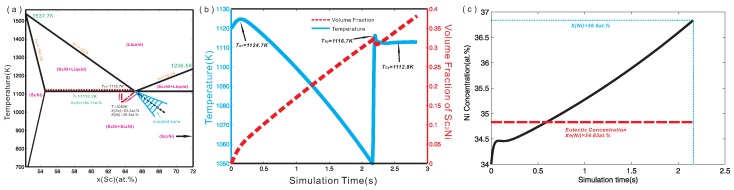
(**a**) Linearized phase diagram of Sc-Ni binary alloy from 52.27 at % Sc to 72 at % Sc constructed according to the recent thermodynamic assessment by Cao et al. [25]; (**b**) Temporal temperature curve (solid-line) and the volume fraction profile (dash-line) during simulation; (**c**) Ni concentration evolution near the Sc${}_{2}$Ni grain.

In the present work, a linearized partial ScNi-Sc${}_{2}$Ni phase diagram was constructed according to the recent thermodynamic assessment of the binary Sc-Ni system by Cao et al. [25], and is displayed in Figure 2a. The melting temperatures of Sc${}_{2}$Ni and ScNi compounds, the liquidus and solidus, as well as the invariant temperatures consistent with the original thermodynamic assessment [25] are also superimposed in Figure 2a. This linearized phase diagram serves as the input of accurate thermodynamic information for quantitative phase-field simulation. Based on the linearized phase diagram, it is known that the extended liquidus of Sc${}_{2}$Ni cannot enter the coupled zone, which provides a possibility for halo formation during solidification [6]. Moreover, the interface energy between liquid and Sc${}_{2}$Ni, liquid and ScNi, as well as that between Sc${}_{2}$Ni and ScNi were set to $1.0\times 10^{-5}$ J/cm${}^{2}$, $1.2\times 10^{-5}$ J/cm${}^{2}$ and $2.0\times 10^{-5}$ J/cm${}^{2}$ respectively. In fact, they were chosen in order to ensure numerical stability during the phase-field simulation. Meanwhile the interface mobility for liquid/Sc${}_{2}$Ni as well as that for liquid/ScNi interface was set as $2.0\times 10^{-2}$ cm${}^{4}$/Js and $1.0\times 10^{-2}$ cm${}^{4}$/Js in order to be in the diffusion-controlled regime. The four-fold anisotropic function [21] was used in the simulation, i.e., $\sigma =\sigma_{0}(1+\delta cos4\theta$). Here, *δ* is the anisotropic coefficient. The anisotropic coefficients of interface energy and interface mobility were set to 0.5 and 0.3 for Sc${}_{2}$Ni while the anisotropic coefficients of interface energy and interface mobility were set to 0.5 and 0.5 for ScNi.

All the phase-field simulations in the present work were carried out in a two-dimensional (2-D) domain (grid-space: 0.2 μm, interface width: 0.8 μm). The domain size was chosen to be 500 × 500 grids. The periodic boundary conditions were set for both phase field and concentration field. Moreover, the heat extraction rate of the simulation system was set to be consistent with the experimental information (200 J/m${}^{3}$). The seed undercooling model incorporated in the MICRESS code was used in the present simulation for modeling the nucleation process. In fact, the seed undercooling model was not a physical one, but was just used for a nucleation criterion based on a user-defined critical undercooling [18]. With the seed undercooling model, a new seed is set if the local undercooling at a nucleant position exceeds a predefined critical nucleation undercooling. The local undercooling depends on the local composition, temperature and curvature. For Sc${}_{2}$Ni phase, only one nucleant position at the center of the simulation domain was set. For ScNi phase, the nucleant positions were set randomly along the interface between liquid and Sc${}_{2}$Ni phase. Using additional parameters like the shield time and distance and the frequency of checking for nucleation, one can design the nucleation conditions which are appropriate for the special requirements [18].

## 3. Results and Discussion

**Figure 3:**
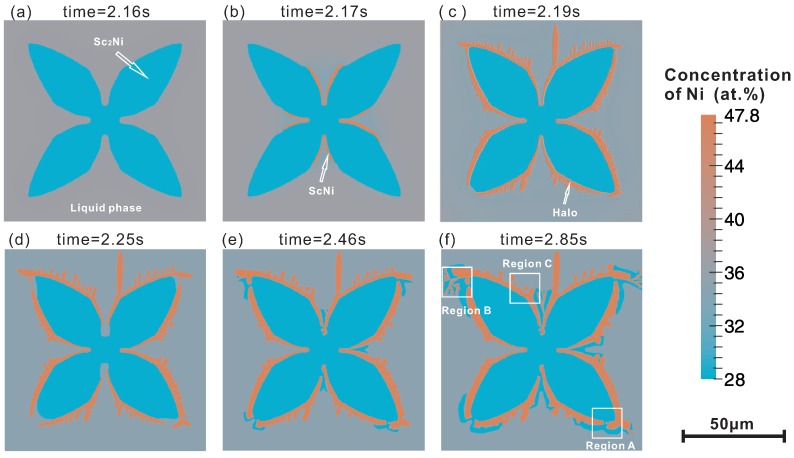
Phase-field simulated microstructure of the Sc-34 at % Ni binary alloy during solidification at different time slides: (**a**) 2.16 s; (**b**) 2.17 s; (**c**) 2.19 s; (**d**) 2.25 s; (**e**) 2.46 s; (**f**) 2.85 s.

Figure 3 shows the phase-field simulated microstructure evolution in the present Sc-Ni binary alloy during solidification with the heat extraction rate 200 J/m${}^{3}$. In Figure 3a–f, the microstructures at different time slides are presented respectively, i.e., 2.16 s, 2.17 s, 2.19 s, 2.25 s, 2.46 s and 2.84 s. According to the simulation results, the microstructure evolution in this ScNi-Sc${}_{2}$Ni off-eutectic alloy can be divided into three stages.

*Stage one:* As can be seen in Figure 3a, the primary Sc${}_{2}$Ni grain grows gradually due to the undercooling. During the initial stage of the solidification, lots of latent heat was released due to the relatively large growth velocity of the Sc${}_{2}$Ni grain. Figure 3b presents the temperature curve (solid line) and the volume fraction profile (dash line) along the simulation time. As can be seen in Figure 3b, the maximum temperature over the simulation can reach 1124.7 K, which is higher than the initial temperature by 4.7 K. This is the first recalescence phenomenon in this ScNi-Sc${}_{2}$Ni off-eutectic alloy during solidification. After the first recalescence, the solute starts to enrich near the tip of the Sc${}_{2}$Ni dendrite, as can be seen in Figure 3c. This can result in a decrease of growth rate of the dendrite. When the latent heat released through the liquid-solid phase transformation is less than the heat extracted from the system, the temperature starts to decrease.

*Stage two:* When the system temperature is lower than the predefined critical nucleation undercooling for the ScNi phase (here, 83 K is set), the secondary phase ScNi can nucleate at the interface of between liquid and Sc${}_{2}$Ni grain, as displayed in Figure 3b. As solidification goes on, the ScNi grains grow fairly quickly, and almost cover the Sc${}_{2}$Ni dendrite, forming a halo structure. The formation of the ScNi halo is due to the relatively large undercooling and growth velocity of ScNi phase. Moreover, as is clearly seen in Figure 3c, several ScNi dendrites appear, and grow into the undercooling melt. However, a large amount of latent heat is released as rapid growth of ScNi particles in an extremely short period (about 0.03 s), which results in the second recalescence phenomenon. Consequently, the system temperature rises up with an extremely large slope (approximate 2223.3 K/s), as can be seen in Figure 2b. Due to the second recalescence phenomenon, the system temperature reaches 1116.7 K, which is higher than the eutectic temperature of the ScNi-Sc${}_{2}$Ni system. With the temperature rising, the Sc${}_{2}$Ni and ScNi dendrites tend to remelt. This can be reflected by the fact in Figure 2b that the volume fraction of Sc${}_{2}$Ni has a slight decrease at the mean time. Moreover, as indicated in Figure 2c, the Ni concentration near the tip of ScNi grains also increases to 36.8 at % on average, at which the two-phase region of liquid and ScNi exists at 1116.7 K. As a result, the solidification sequence changes as the dashed line indicates in Figure 1. Then, the ScNi grains grow as the second primary phase with dendrite structure, as shown in Figure 3d.

*Stage three:* In this stage, the temperature drops to an approximate equilibrium value (1112.8 K) which is slightly below the eutectic temperature (1113.3 K). The first primary phase Sc${}_{2}$Ni, the second primary phase ScNi and the Sc${}_{2}$Ni-ScNi lamellar eutectic structure grow simultaneously. As can be seen in Figure 3e,f, Sc${}_{2}$Ni grows through the gap between two neighboring ScNi grains and surround the ScNi dendrites in the marked “region A”. Meanwhile, the lamellar eutectic structure appears during the coupled growth of Sc${}_{2}$Ni and ScNi, corresponding to the marked “region B”. Furthermore, the necking and detachment phenomenon appear in some branches of the ScNi dendrites, marked as region “C” due to the effect of curvature and microsegregation [18]. The grains detached from the ScNi dendrites will grow as round isolated islands and distribute in the liquid phase homogeneously.

As indicated above, the present phase-field simulation can well reproduce the experimental microstructure. Likewise, the phase-field simulation can also nicely reveal the mechanism for the anomalous halo formation in the present ScNi-Sc${}_{2}$Ni off-eutectic binary alloy. In the relatively small arc-melted sample, no noticeable temperature gradient exits. Thus, the latent heat released during solidification cannot be transported out immediately and may influence the local sample temperature field. The released latent heat causes the recalescence phenomenon and changes the solidification sequence accordingly, resulting in such anomalous halo structure.

## 4. Conclusions

In summary, an anomalous halo formation was observed in an arc-melted ScNi-Sc${}_{2}$Ni off-eutectic binary alloy. Considering that the previously proposed mechanisms cannot give good explanations, the quantitative phase-field simulation was utilized to reproduce the microstructure evolution in the target alloy. Based on the phase-field simulation, one knows that (i) the large undercooling and growth velocity of the ScNi phase during solidification cause the formation of halos; and (ii) the released latent heat induces the recalescence phenomenon, and changes the solidification sequence largely, resulting in the anomalous halo structure in the Sc-34 at % Ni alloy.
